# The role of OIP5 in the carcinogenesis and progression of ovarian cancer

**DOI:** 10.1186/s13048-023-01265-4

**Published:** 2023-09-02

**Authors:** Xin Zhang, Wenjie Gu, Aiqin Lin, Renjie Duan, Likai Lian, Yuanyuan Huang, Tiechen Li, Qing Sun

**Affiliations:** 1 Department of Gynecology and Obstetrics, Yijishan Hospital of Wannan Medical College, Wuhu, Anhui P.R. China; 2 School of Basic Medical Sciences, Wannan Medical College, Wuhu, Anhui P.R. China

**Keywords:** OIP5, Ovarian cancer, Proliferation, Migration and invasion, Cell cycle, Apoptosis

## Abstract

**Background:**

Opa interacting protein 5 (OIP5), which is a cancer/testis-specific gene, plays a cancer-promoting role in various types of human cancer. However, the role of OIP5 in the carcinogenesis and progression of ovarian cancer remains unknown.

**Methods:**

We first analyzed the expression of OIP5 in ovarian cancer and various human tumors with the Sangerbox online analysis tool. GSE12470, GSE14407 and GSE54388 were downloaded from the Gene Expression Omnibus (GEO) database, and GEO2R was used to screen differentially expressed genes in ovarian cancer tissues. Gene Ontology (GO) enrichment analysis was used to explore the related biological processes. Receiver operating characteristic (ROC) curve was generated to evaluate the predictive ability of OIP5 for ovarian cancer. Next, RT–PCR, immunohistochemistry and Western blotting were utilized to evaluate the expression of OIP5 in ovarian cancer. CCK8, EdU proliferation assays and colony formation assays were used to measure cell proliferation, cell cycle progression was examined by PI staining and flow cytometry, and cell apoptosis was examined by Caspase3/7 activity assays. The effect of OIP5 on the migration and invasion of ovarian cancer cells was analyzed with Transwell assays.

**Results:**

We found that OIP5 is highly expressed in ovarian cancer through bioinformatics analysis, and importantly, OIP5 may be an important biomarker for the prognosis and diagnosis of ovarian cancer. RT–PCR assays, immunohistochemistry and Western blotting were also used to confirm the high expression of OIP5 in ovarian cancer. Subsequently, we demonstrated that the proliferation and migration of the ovarian cancer cell line A2780 were significantly inhibited after OIP5 gene silencing, apoptosis was increased and cell cycle progression was arrested at the G1 phase.

**Conclusion:**

This study indicated that OIP5 was highly expressed in ovarian cancer and that downregulation of OIP5 inhibited the proliferation, migration and invasion of ovarian cancer cells, induced cell cycle arrest and promoted cell apoptosis. Therefore, OIP5 may be an important biomarker for the early diagnosis and potential target for treatment of ovarian cancer.

**Supplementary Information:**

The online version contains supplementary material available at 10.1186/s13048-023-01265-4.

## Introduction

Ovarian cancer (OV) is a common malignant tumor in women, and patients with ovarian cancer have a five-year survival rate of less than 50% [1]. According to the cancer statistics from China and the United States in 2022, the incidence rate of ovarian cancer ranks fourth after breast cancer, cervical cancer and uterine body cancer, while its mortality rate ranks first [2]. Ovarian cancer is a malignant tumor that seriously threatens women’s health. Ovarian cancer is often referred to as silent killer because of the lack of obvious symptoms in its early stages, and over 70% of ovarian cancer patients are not diagnosed until the disease has progressed to stage III or IV [1, 3]. This largely explains the poor prognosis of ovarian cancer patients. At present, surgery combined with platinum chemotherapy drugs is still the initial treatment method for ovarian cancer, but most patients experience successive relapse after treatment [4, 5]. Therefore, it is particularly important to explore new diagnostic and prognostic biomarkers to improve the early diagnosis and treatment of ovarian cancer.

Opa interacting protein 5 (OIP5) is a member of the cancer/testis antigen (CTA) family. It is located on human chromosome 15q15 and consists of five exons. Its mRNA sequence is 1249 bp long and encodes the OIP5 protein, which has a molecular weight of 25 kDa [6]. OIP5 plays an important role in maintaining the structure and function of centromeres [7]. Studies have shown that the OIP5 gene plays an important role in regulating cell cycle progression through the E2F-2b pathway by interacting with retinoblastoma protein and lamina-associated polypeptide 2ɑ (LAP2ɑ) [8, 9]. Abnormal expression of OIP5 has been identified in several tumors, such as breast cancer [10], glioblastoma [11], hepatocellular carcinoma [12]and bladder cancer [13]. For example, overexpression of OIP5 can promote the proliferation of liver cancer cells and inhibit their apoptosis [14]. The increased expression of OIP5 is also significantly associated with poor prognosis of lung cancer and esophageal cancer, and it is also a prognostic biomarker and a potential target for treatment [15]. However, at present, a detailed understanding of the role of OIP5 in ovarian cancer is lacking. Hence,we proposed a scientific hypothesis to determine whether the OIP5 gene can also promote tumor progression and metastasis in ovarian cancer. This study may provide new prospects for the diagnosis and treatment of ovarian cancer.

## Results

### Bioinformatics analysis of OIP5 expression in ovarian cancer

**Fig. 1:**
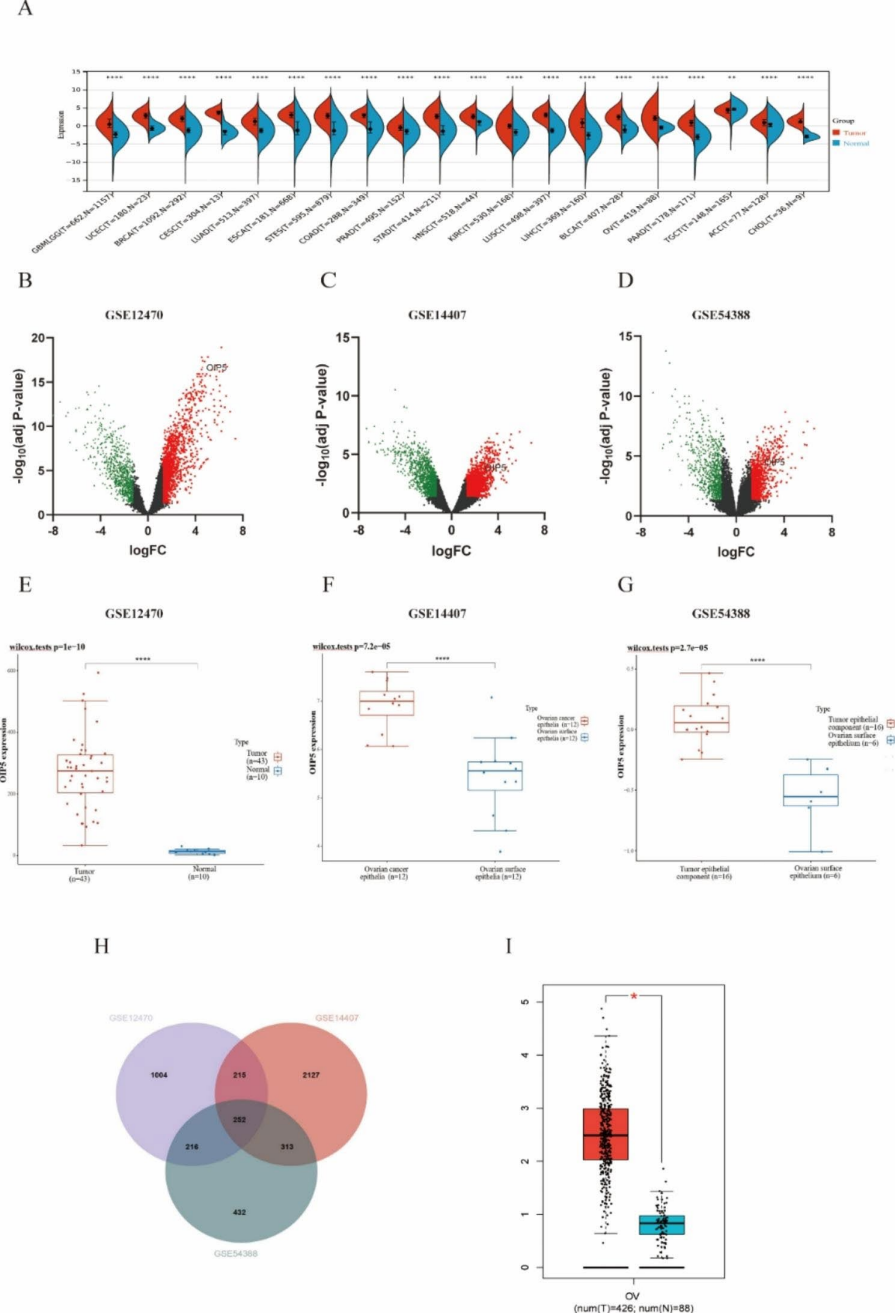
Identification of differentially expressed genes (DEGs) based on bioinformatics analysis. **A** The expression of OIP5 in various human cancers compared with normal tissues. **B-D** Volcano plots of DEGs in the GSE12470 (n = 53), GSE14407 (n = 24) and GSE54388 (n = 22) datasets in ovarian cancer. **E-G** OIP5 was highly expressed in ovarian cancer in three databases. **H** Venn diagram showing the number of DEGs that were upregulated in the three datasets. **I** The mRNA expression of OIP5 in ovarian cancer was further verified by the GEPIA database. GEPIA, Gene Expression Profiling Interactive Analysis. *P < 0.05, ****P < 0.0001

**Fig. 2:**
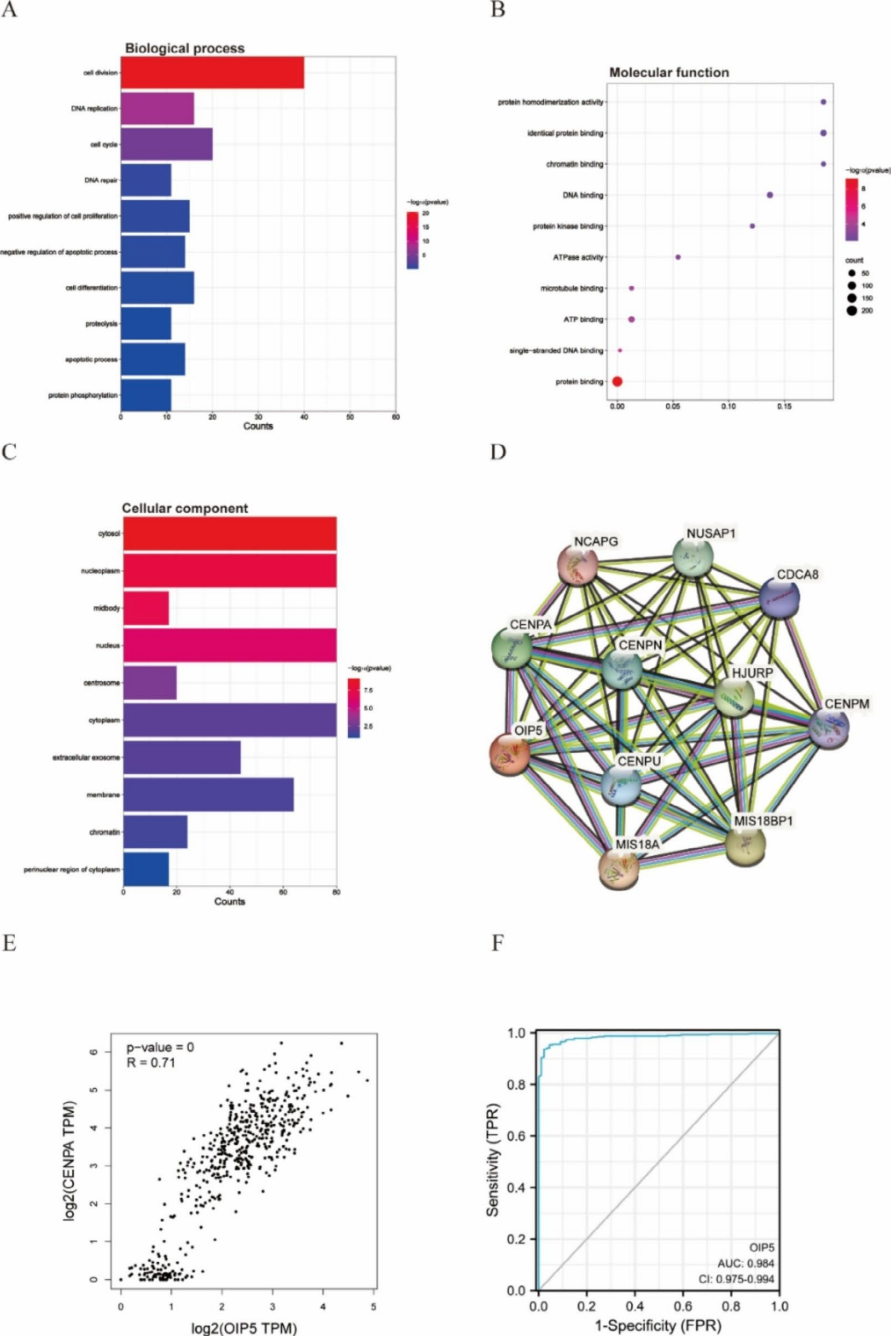
Enrichment analysis of DEGs in ovarian cancer and constructing a PPI network related to OIP5. A-C Enrichment analysis of GO terms for differentially expressed genes. GO, Gene Ontology. **A** Biological process; **B** Molecular function; **C** Cellular component. **D** The PPI network related to OIP5 was obtained through the STRING platform. PPI, Protein‒Protein Interaction. **E** The correlation between OIP5 and CENPA expression was determined with the GEPIA database. CENPA, Centromere protein A. **F** ROC curve verified the sensitivity and specificity of OIP5 as a prognostic biomarker of ovarian cancer. The closer the area under the curve (AUC) is to 1, the better the diagnostic effect. ROC, Receiver operating characteristic

We first evaluated the expression of OIP5 in pan-cancer data from TCGA and GTEx. As shown in Fig. 1A, OIP5 was significantly overexpressed in 19 cancer samples, including GBMLGG, UCEC, BRCA, CESC, LUAD, ESCA, STES, COAD, PRAD, STAD, HNSC, KIRC, LUSC, LIHC, BLCA, OV, PAAD, ACC, and CHOL samples, compared with normal samples. In contrast, its expression was downregulated in TGCT. As shown in Fig. 1B-D, the volcano plot shows the differentially expressed genes (DEGs) in the GSE12470, GSE54388 and GSE14407 datasets, where red represents upregulated genes and green represents downregulated genes. High expression of OIP5 was observed in ovarian cancer samples in three datasets (Fig. 1E-G). In addition, a Venn diagram revealed the genes with upregulated expression in the three datasets, and 252 genes were identified, including OIP5 (Fig. 1H). Then, we further verified the high expression of OIP5 in ovarian cancer with the GEPIA database (Fig. 1I). GO enrichment analysis demonstrated that 252 genes were mainly enriched in cell division, DNA replication, and cell cycle in the biological process category (Fig. 2A); protein binding and ATP binding in the molecular function category (Fig. 2B); and the cytosol, nucleoplasm and nucleus in the cellular component category (Fig. 2C). We observed a functional interaction between OIP5 and CENPA by establishing a PPI network (Fig. 2D). Centromere protein A (CENPA) plays a key role in regulating cell cycle progression and cell division. Next, we further investigated the positive correlation between OIP5 and CENPA in ovarian cancer by GEPIA (Fig. 2E). Eventually, the ROC curve verified the sensitivity and specificity of OIP5 as a biomarker for the diagnosis of ovarian cancer, with an area under the curve (AUC) equal to 0.984 (CI: 0.975–0.994) (Fig. 2F).

### OIP5 was upregulated in ovarian cancer cell lines and tissues

**Fig. 3:**
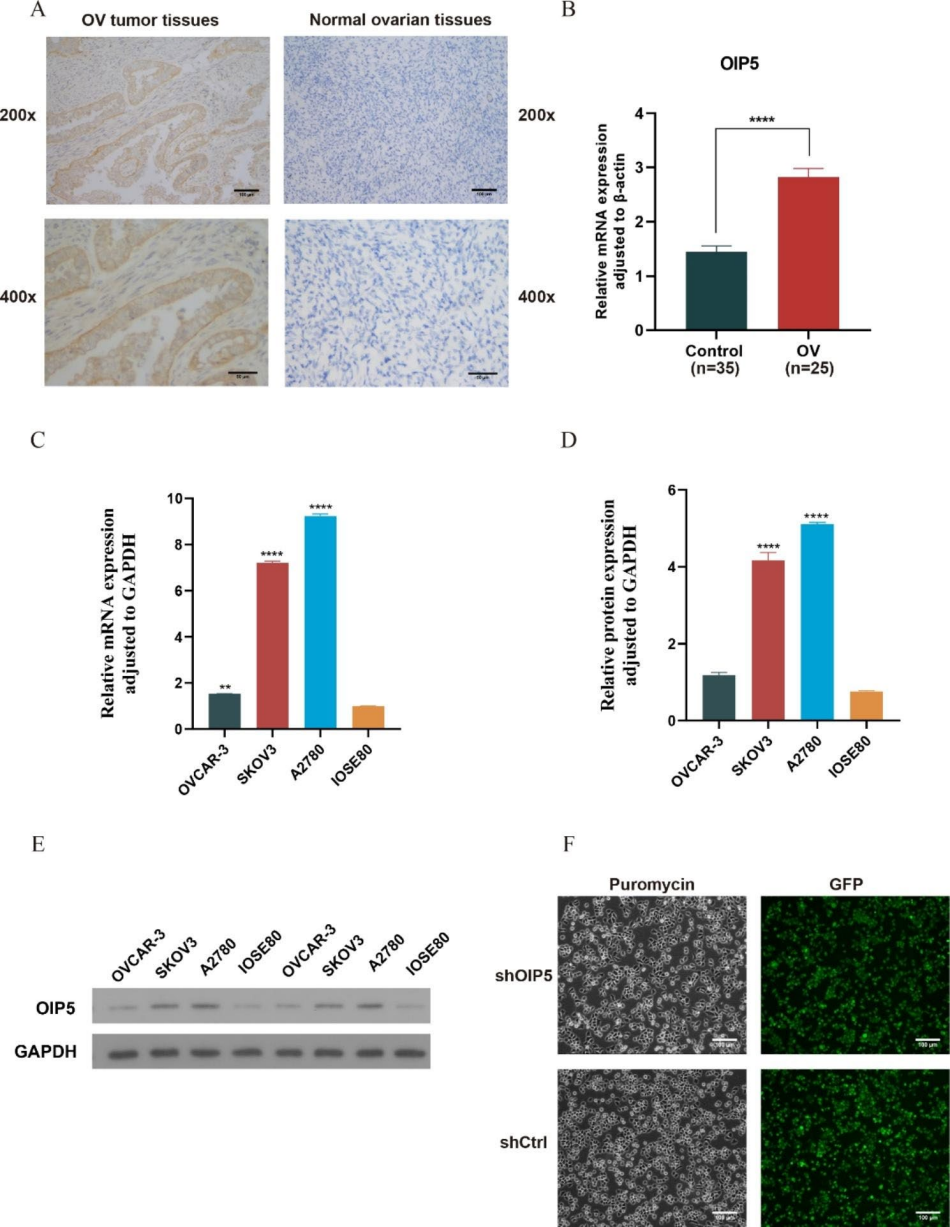
The expression of OIP5 in ovarian cancer tissues and cells and detection of siRNA lentivirus infection. **A** IHC staining was used to analyze the protein expression levels of OIP5 in ovarian cancer tissues (n = 15) and normal ovarian tissues (n = 13) (magnification, 200x or 400x; scale bar, 100 μm or 50 μm). IHC, Immunohistochemistry. **B** The mRNA expression of OIP5 in ovarian cancer tissues (n = 25) and normal ovarian tissues (n = 35). **C** The mRNA expression of OIP5 in ovarian cancer cell lines. **D** and **E** Protein expression of OIP5 in ovarian cancer cell lines. **F** Puromycin resistance screening to identify A2780 cells infected with lentivirus. GFP detection to assess lentivirus infection in A2780 cells (scale bar, 100 μm). GFP, Green Fluorescent Protein. **P < 0.01, ****P < 0.0001

We analyzed the protein expression of OIP5 in ovarian cancer and normal ovarian tissues through immunohistochemistry. We found that the expression of OIP5 was significantly upregulated in ovarian cancer tissues compared to normal ovarian tissues (Fig. 3A). RT‒PCR was used to analyze the mRNA expression level of OIP5 in ovarian cancer tissues and cell lines. The results showed a significant increase in the mRNA expression level of OIP5 in ovarian cancer compared to normal ovarian tissues (Fig. 3B). Moreover, the mRNA expression of OIP5 in ovarian cancer cell lines (SKOV3, A2780, OVCAR-3 cells) was significantly higher than that in the IOSE80 normal ovarian cell line (Fig. 3C). We also analyzed the protein expression of OIP5 in ovarian cancer cell lines using western blotting. The results showed that the protein expression of OIP5 was significantly increased in three ovarian cancer cell lines (SKOV3, A2780, OVCAR-3 cells) compared with IOSE80 cells (Fig. 3D-E). Among them, OIP5 had a higher expression level in the A2780 cell line than in the SKOV3 and OVCAR-3 cell lines. In summary, these results indicated that OIP5 was highly expressed in ovarian cancer tissues and cell lines. For this reason, siRNA lentivirus infection was used to knock down the expression of OIP5 in A2780 cells. The knockdown efficiency of OIP5 was detected through RT‒PCR (Fig. S1). As shown in Fig. 3F, puromycin resistance screening and green fluorescent protein (GFP) detection showed that siRNA lentivirus infection of A2780 cells was successful.

### Knockdown of OIP5 inhibited the proliferation of ovarian cancer cells

**Fig. 4:**
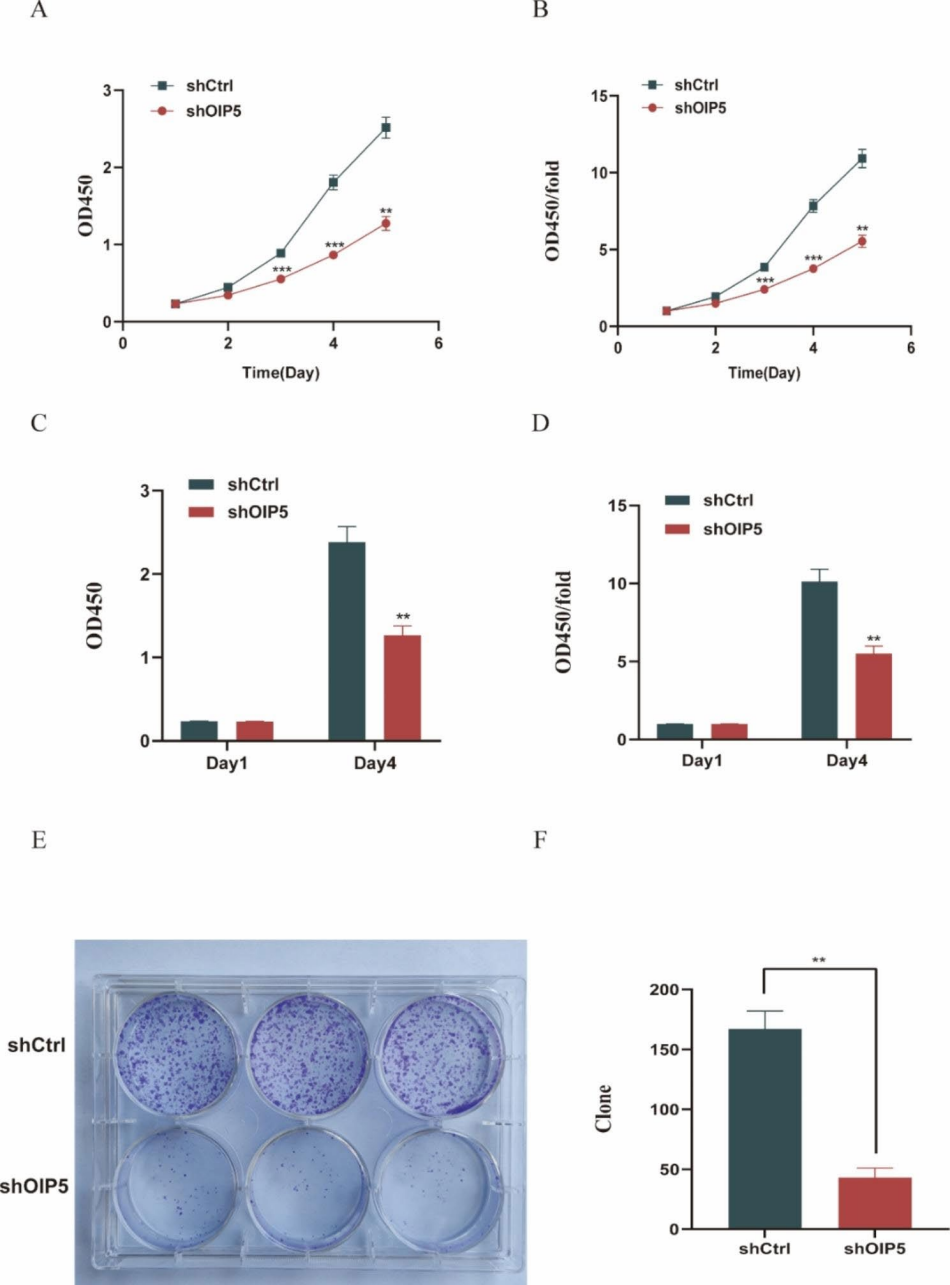
Knockdown of OIP5 inhibits proliferation and colony formation in ovarian cancer cells. **A** CCK8 assay was used to measure the change in the OD450 value from Day 1 to Day 5. **B** The OD450/fold is a multiple of the OD450 value of Day 1 to Day 5 relative to Day 1. The CCK8 assay showed that knockdown of OIP5 significantly inhibited the proliferation of ovarian cancer cells. CCK8, Cell counting kit-8. **C** EdU was used to measure the OD450 value on Day 1 and Day 4. **D** The OD450/fold is the multiple of the OD450 value of Day 4 relative to Day 1. EdU proliferation assays showed that OIP5 knockout significantly inhibited the proliferation of ovarian cancer cells. EdU, 5-Ethynyl-2-deoxyuridine. **E** and **F** Clone formation assays showed that knockdown of OIP5 suppressed colony formation ability. **P < 0.01, ***P < 0.001

The CCK8 assay and EdU proliferation assay were used to determine the role of OIP5 in A2780 cell proliferation. The experimental results indicated that the proliferation of A2780 cells was significantly reduced after knockdown of OIP5 gene expression (Fig. 4A-D), and OIP5 knockdown also markedly inhibited the proliferation of SKOV3 cells through the CCK8 assay (Fig. S2). In addition, the colony formation assay results showed that the downregulation of OIP5 significantly inhibited the colony formation ability of A2780 cells (Fig. 4E-F). In conclusion, these results indicated that knockdown of OIP5 significantly inhibited the proliferation of ovarian cancer cells.

### Knockdown of OIP5 suppressed the migration and invasion of ovarian cancer cells

**Fig. 5:**
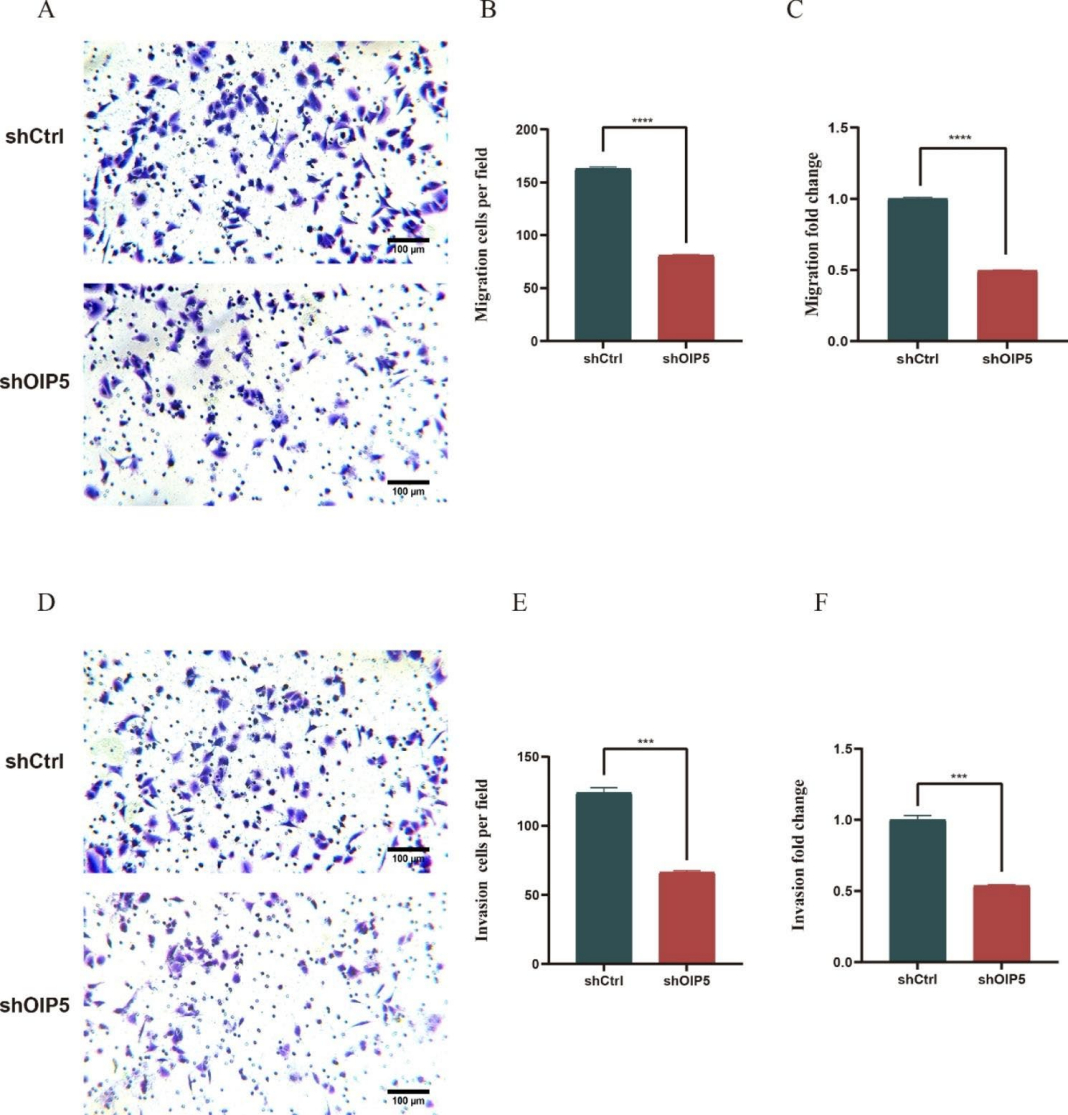
Knockdown of OIP5 suppresses the migration and invasion of ovarian cancer cells. **A** OIP5 silencing significantly inhibited the migration of ovarian cancer cells. OIP5, Opa interacting protein 5. **B** Cell counting was performed on migrating cells. **C** The numbers of migrating cells in the shCtrl samples were set to 1, and the fold change was determined for the shOIP5 samples. **D** OIP5 silencing significantly inhibited the invasion of ovarian cancer cells. **E** Cell counting was performed on invading cells. **F** The numbers of invading cells in the shCtrl samples were set to 1, and the fold change was obtained for the shOIP5 samples. ***P < 0.001, ****P < 0.0001

Next, a Transwell assay was used to determine the effect of OIP5 on the migration and invasion of A2780 ovarian cancer cells. The results showed that the migration and invasion of A2780 ovarian cancer cells were significantly decreased after OIP5 silencing (Fig. 5A-F). In addition, OIP5 knocking down also significantly suppressed the migration and invasion of OVCAR-3 cells (Fig. S3A-F). In short, these results revealed that OIP5 promoted the migration and invasion of ovarian cancer cells, and knockdown of OIP5 markedly suppressed the migratory and invasive abilities of ovarian cancer cells.

### Downregulation of OIP5 induces cell cycle arrest and apoptosis in ovarian cancer cells

**Fig. 6:**
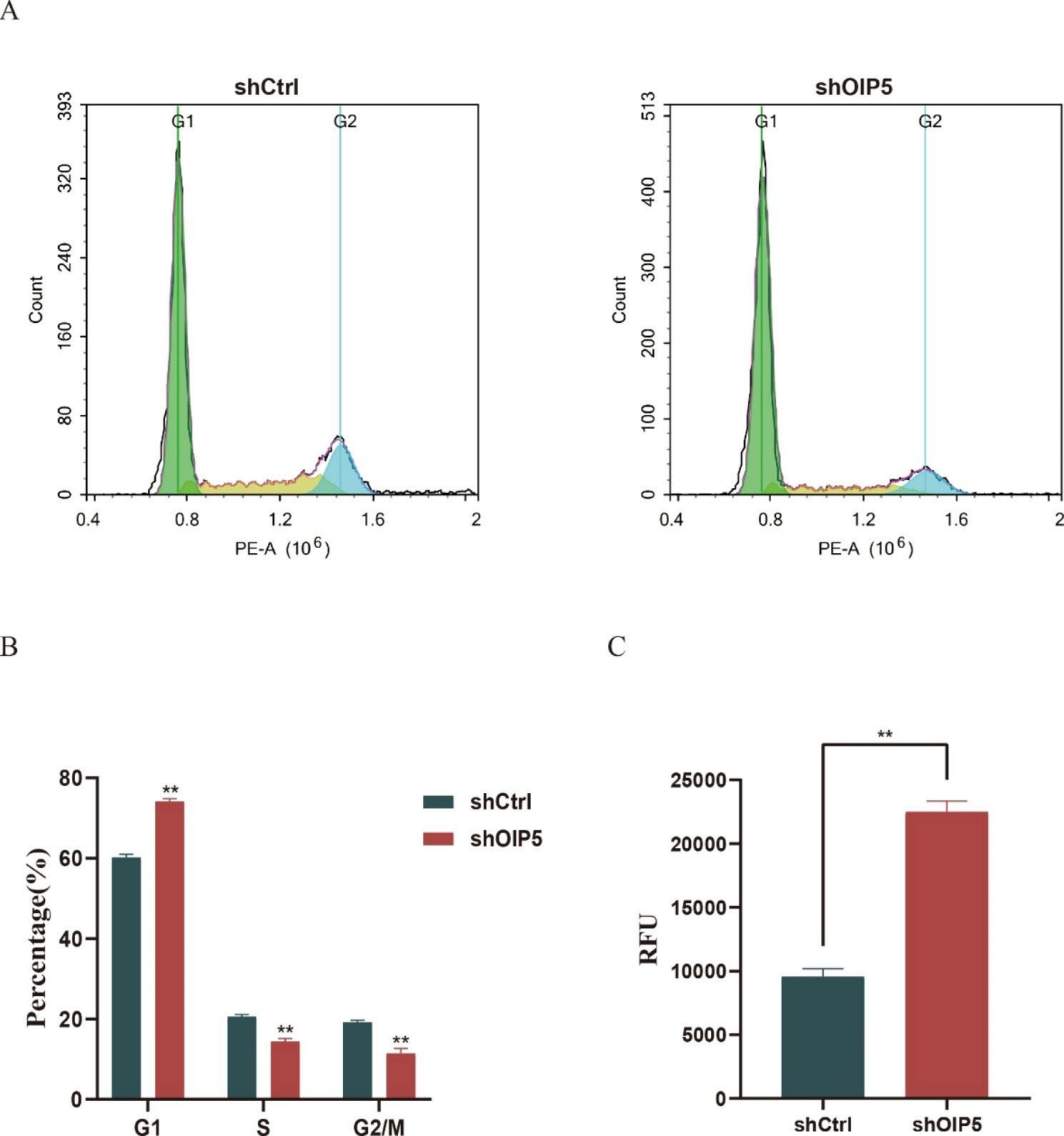
Knockdown of OIP5 induces cell cycle arrest and apoptosis in ovarian can-cer cells. **A** and **B** Cell cycle distribution showed that OIP5 knockdown caused an increase in the number of cells in the G1 phase and a decrease in the number of cells in the S phase and G2/M phase among ovarian cancer cells. This indicated that the downregulation of OIP5 stalled cell cycle progression in the G1 phase. OIP5, Opa interacting protein 5. **C** OIP5 knockdown induced apoptosis in ovarian cancer cells. RFU, relative fluorescence units. **P < 0.01

Flow cytometry was used to determine the effect of OIP5 on cell cycle progression. The percentage of A2780 ovarian cancer cells in the G1 phase was significantly increased after OIP5 silencing, while the percentage of cells in the S phase and G2/M phase was decreased. This indicated that the downregulation of OIP5 stalled cell cycle progression in the G1 phase (Fig. 6A-B). Subsequently, cell apoptosis after OIP5 knockdown was measured by measuring caspase-3/7 activity. The results showed that downregulation of OIP5 resulted in increased apoptosis of A2780 ovarian cancer cells (Fig. 6C). Taken together, these results indicated that downregulation of OIP5 induced cell cycle arrest and apoptosis in ovarian cancer cells.

## Discussion

Ovarian cancer is the deadliest malignant tumor in the field of gynecology. Currently, surgery and chemotherapy are the main treatment methods for cancer, but most patients relapse due to chemotherapy resistance within a few years after initial treatment [2–4]. The malignant transformation of ovarian epithelial cells into cancer cells is an extremely complex process involving multiple genes and pathways [16, 17]. Therefore, it is critical to explore the molecular mechanism of ovarian cancer carcinogenesis and progression and to search for specific biomarkers to provide a new target for clinical treatment [18]. OIP5 is a cancer/testis-specific antigen, and it has been reported that OIP5 plays a key role in cell cycle regulation. Abnormal expression of genes in cancer lead to tumorigenesis [19, 20]. Previous studies have shown that OIP5 is highly expressed in various human malignant tumors, such as breast cancer [10], malignant glioma [21], liver cancer [14], and bladder cancer [22], and can cause corresponding immune responses in tumor patients. Gong et al. confirmed that OIP5 is related to the infiltration of T cells, B cells, macrophages and other immune cells in renal clear cell carcinoma and is a potential immunotherapeutic target for renal clear cell carcinoma [23]. Furthermore, OIP5 is also involved in regulating the proliferation, migration, invasion, apoptosis and other biological behaviors of tumor cells, thus promoting the occurrence and development of tumors. For example, Zheng et al. found that OIP5 is highly expressed in nasopharyngeal carcinoma, and its knockdown can inhibit epithelial mesenchymal transformation (EMT) by regulating JAK2/STAT3 signaling pathway, thereby inhibiting the metastasis of nasopharyngeal carcinoma cells [24]. Wang et al. confirmed from in vivo and in vitro experiments that the upregulation of OIP5 promotes the proliferation, metastasis and drug resistance progression of bladder cancer, and it has become a potential therapeutic target and biomarker of bladder cancer prognosis [25]. Li et al. analyzed the signaling pathway downstream of OIP5 through the proteomic kinase spectrum and found that upregulation of OIP5 can induce AKT activation through the mammalian target of rapamycin protein complex 2 (mTORC2), and P38/phosphatase and tensin homologous signal pathways, and enhance its nuclear translocation and promote tumor progression through the phosphorylation of β- catenin and glycogen synthase kinase-3β (GSK-3β). The downregulation of OIP5 was achieved by regulating mTORC1 and the β- catenin signaling pathway inhibited the growth and metastasis of hepatocellular carcinoma [12]. However, the role of OIP5 in the carcinogenesis and progression of ovarian cancer has not been investigated.

In this study, we found that OIP5 is highly expressed in ovarian cancer and is closely related to the cell cycle through bioinformatics analysis. In addition, immunohistochemistry, RT‒PCR, and Western blotting were used to further validate the high expression of OIP5 in ovarian cancer. Subsequently, the OIP5 gene was knocked down by siRNA lentivirus infection. Cell function assays confirmed that knockdown of OIP5 significantly suppressed the proliferation, migration and invasion of A2780 ovarian cancer cells, apoptosis was increased, and the cell cycle was arrested at the G1/S phase. These results suggested that OIP5 may function as an oncogene in the carcinogenesis and progression of ovarian cancer. However, this study has some limitations that could be addressed by increasing the number of ovarian cancer cell lines and further exploring the mechanism of OIP5 in the carcinogenesis and progression of ovarian cancer.

## Conclusions

In summary, OIP5 was highly expressed in ovarian cancer, and knockdown of OIP5 significantly inhibited the proliferation, migration and invasion of ovarian cancer cells; cell apoptosis increased, and the cell cycle was arrested in the G1/S phase when the OIP5 gene was silenced. The current research results suggest that OIP5 may be a meaningful biomarker for the early diagnosis and treatment of ovarian cancer.

## Methods

### Data source and analysis of differential expression

First, the Sangerbox (www.sangerbox.com) online tool was used to analyze the expression of OIP5 in ovarian cancer and various human tumors. The ovarian cancer datasets GSE12470, GSE14407 and GSE54388 were all from the Gene Expression Omnibus (GEO) database (https://www.ncbi.nlm.nih.gov/gds). The GEO2R data analysis tool was used to screen differentially expressed genes (DEGs) between normal ovarian tissue specimens and ovarian cancer specimens. P < 0.05 and |logFC|>1.2 were used as criteria for screening DEGs. Volcano plots showing up- and downregulation of DEGs were generated. In addition, mRNAs with different expression in three datasets were overlapped with Evenn (www.ehbio.com/test/venn/#/). We analyzed transcriptome data from The Cancer Genome Atlas (TCGA) and Genotype-Tissue Expression (GTEx) databases through the Gene Expression Profiling Interactive Analysis (GEPIA) (http://gepia.cancer-pku.cn/index.html) bioinformatics website to further verify the expression of the OIP5 gene in ovarian cancer. Subsequently, Gene Ontology (GO) enrichment analysis was performed using the DAVID online tool (https://david.ncifcrf.gov). STRING (https://string-db.org) was used to construct protein‒protein interaction (PPI) networks. Next, GEPIA was used to further evaluate the correlation between OIP5 and Centromere protein A (CENPA) expression. Finally, RNAseq data from TCGA and GTEx transcripts per million reads (TPM) format were uniformly processed by the Toil process of UCSC XENA (https://xenabrowser.net/datapages/) and comparison of expression between samples was performed after log2 transformation of RNAseq data. Receiver operating characteristic (ROC) curve analysis was performed by using R software (version 3.6.3), the pROC package [version 1.17.0.1] (for analysis) and the ggplot2 package [version 3.3.3] (for visualization) to determine the specificity and sensitivity of OIP5 as a predictive biomarker. Among them, the horizontal axis of the curve represents the false-positive rate (FPR), and the vertical axis represents the true positive rate (TPR). The closer the area under the curve (AUC) is to 1, the better the diagnostic effect.

### Patient and tissue samples

Tumor tissues were collected from 28 ovarian cancer patients who underwent gynecological and obstetric surgeries at Yijishan Hospital affiliated with Wannan Medical College from July 2019 to July 2021, and normal ovarian tissues were collected from patients who underwent total hysterectomy and unilateral adnexectomy or ovarian biopsy. All fresh tissue samples were immediately placed into a -80 ℃ freezer. This work was approved by the Ethics Committee of the Yijishan Hospital Affiliated to Wannan Medical College, and written informed consent was obtained from the patients.

### Cell lines and cell cuture

The human SKOV3 and A2780 ovarian cancer cell lines and the IOSE80 normal ovarian epithelial cell line were purchased from Wuhan Procell Life Science&Technology (Wuhan, China). The OVCAR-3 cell line was obtained from the Cell Resource Center of Shanghai Institute of Life Sciences Chinese Academy of Sciences (Shanghai, China). The SKOV3 cell line was cultured in McCoy’s 5 A medium (Gibco, Carlsbad, CA, USA), the A2780 cell line was cultured in 1640 medium (Gibco, Carlsbad, CA, USA), and the IOSE80 cell line was cultured in DMEM (Gibco, Carlsbad, CA, USA). The three cell lines were cultured in medium supplemented with 10% fetal bovine serum (Gibco, Carlsbad, CA, USA) and 1% penicillin and streptomycin. The OVCAR-3 cell line was cultured in 1640 medium (Gibco, Carlsbad, CA, USA) supplemented with 20% fetal bovine serum and 0.01 mg/ml insulin. The cells were cultured in an incubator at 37 °C in 5% CO_2_.

### Reverse transcription quantitative polymerase chain reaction (RT‒PCR)

Total RNA was extracted from ovarian cancer cells and tissues by using TRIzol reagent (Invitrogen, Carlsbad, CA, USA). The RNA was reverse transcribed to cDNA using SuperScript™ III First-Strand Synthesis SuperMix for qRT‒PCR (Invitrogen, Carlsbad, CA, USA) for 10 min at 60 °C, 30 min at 50 °C and 5 min at 85 °C according to the instructions provided by the kit. qRT‒PCR was performed using SYBR Green PCR Master Mix (Applied Biosystems) on a CFX384 Real-Time fluorescence quantitative PCR Detection system (Bio-Rad Laboratories, Inc.). OIP5 mRNA expression was quantified by the 2^−ΔΔCT^ method and standardized to the internal reference genes GAPDH and β-actin. All the primer sequences in this study are presented in Table 1.

**Table 1:** The primers sequences for RT-PCR

Gene	Forward primer(5´-3´)	Reverse primer(5´-3´)
OIP5	CCCTTCCTAGTTGGCATTGA	GCACACCATTTTGTCACTGG
GAPDH	CCATGACAACTTTGGTATCGTGGAA	GGCCATCACGCCACAGTTTC
β-actin	GTCATTCCAAATATGAGATGCGT	GCTATCACCTCCCCTGTGTG

### Immunohistochemistry assay

For the immunohistochemistry assay, the tissues were fixed with 4% paraformaldehyde, incubated in liquid paraffin, and cut into 5 μm sections using a paraffin slicer. Then, the sections were dewaxed in xylene and hydrated in an alcohol series. The sections were incubated in 3% H_2_O_2_ to inhibit endogenous peroxidase activity. Subsequently, the sections were incubated with rabbit polyclonal anti-OIP5 antibody overnight at 4 °C. After washing with PBS, the sections were incubated with biotin-labeled secondary antibody at room temperature for 1 h. Finally, the sections were stained with diaminobenzidine (DAB), and hematoxylin was used for restaining. Protein staining was analyzed using a microscope.

### Western blotting

Total protein was harvested from ovarian cancer cells using the total protein extraction kit (including Protease Inhibitor Cocktail). The protein concentration was determined with a BCA protein assay kit (Beyotime, Haimen, China). The protein samples were separated on a 10% SDS‒PAGE gel and transferred to PVDF membranes (Millipore, United States). Then, the membranes were blocked with 5% BSA (Sangon Biotech, Shanghai, China) at room temperature for at least 1 h. After incubation with anti-OIP5 antibody (cat. no. HPA052271; Sigma, 1:500) and anti-GAPDH antibody (cat. no. ab181602; Abcam, 1:10000) overnight at 4, the membranes were incubated with anti-mouse or anti-rabbit (cat.no. A0216 and A0208; Beyotime; 1:3000) secondary antibodies conjugated with horseradish peroxidase at room temperature for 1 h and washed three times with TBS-T. Finally, SuperSignal West Dura Extended Duration Substrate (Thermo, Waltham, MA, USA) was used to detect the proteins on the membranes.

### Lentivirus transfection

OIP5 was knocked down in A2780 cells by using a lentivirus vector. The lentivirus targeting sequence was designed and synthesized by Shanghai Gemma Gene Co., Ltd. A2780 cells were infected with lentivirus and cultured in 1640 medium supplemented with 10% FBS, and 8 μg/μl polybrene was added. Purinomycin at 2 μg/ml was added to the medium 72 h after infection. The medium was replaced with new medium every two days, supplemented with puromycin, and continuously screened until there was no obvious cell death. The fluorescent images were captured 96 h after infection. The design results of OIP5 lentivirus interference target were presented in Supplementary material Table S1.

### Cell counting kit-8 (CCK-8) assay

A2780 cells infected with shOIP5 or shCtrl were seeded in 96-well plates at a density of 4000 cells per well in 100 μl. The cells were cultured in an incubator at 37 °C in 5% CO_2_. Next, 10 μl of CCK-8 solution (cat. no. BL001B500T; Biomiky) was added to each well at the specified time points (24 h, 48 h, 72 h, or 96 h). The A2780 cells were incubated at 37 °C for 2 h. The absorbance values were measured at 450 nm by a microplate reader (Bio-Rad, Hercules, CA).

### EdU proliferation assay

A2780 cells infected with shOIP5 or shCtrl were seeded in 96-well plates at a density of 2000 cells per well in 100 μl. EdU reagent (Sangon Biotech, Shanghai, China) was added, and the cells were incubated in a 37 °C incubator for 2 h on the first day and the fourth day after seeding. Click reaction solution was added to each well and incubated at room temperature in the dark for 30 min. Subsequently, streptavidin HRP working solution was added to each well and incubated at room temperature for 30 min. The absorbance values were determined at 370/620–650 nm.

### Colony formation assay

A2780 cells infected with shOIP5 or shCtrl were seeded in 6-well plates at a density of 600 cells per well, and the cells were continuously cultured in 5% CO_2_ at 37 °C for 14 days. Cell colonies were fixed with 4% paraformaldehyde for 20 min, and crystal violet dye was added and incubated for 10 min. Clone counting was performed after washing with PBS and drying at room temperature. Then, photos were taken with a camera.

### Transwell assays

A2780 cells infected with shOIP5 or shCtrl were seeded in the upper chamber of 24-well plates at a density of 5х10^4^ cells per well. Then, 600 μl of 1640 medium supplemented with 10% fetal bovine serum (FBS) was added to the lower chamber. The cells were incubated in a 37 °C incubator for 48 h. Then, the cells in the upper chamber were gently removed with a cotton swab and washed three times with PBS. The cells in the bottom chambers were fixed with 4% paraformaldehyde for 30 min and stained with 0.1% crystal violet (cat. no. C0775; Sigma) for 20 min at room temperature. For the invasion assay, the cells were seeded in the upper chamber coated with Matrigel (BD, Biosciences, USA) at a density of 6х10^4^ cells per well. The chamber was cultured in an incubator for 48 h. Five visual fields were randomly selected under the microscope, and cell migration and invasion were determined by counting the number of cells.

### Cell cycle analysis

A2780 cells infected with shOIP5 or shCtrl were seeded in 6-well plates at 3х10^5^ per well. When the cell density reached 80%, the cells were collected and centrifuged at 13,000 rpm for 5 min and fixed with ethanol for at least 1 h. Then, the cells were stained with propidium iodide (PI) solutions for 30 min in the dark at 37 ℃. Red fluorescence was detected at 488 nm by using flow cytometry (BD, Biosciences, USA).

### Caspase3/7 activity assay

A2780 cells infected with shOIP5 or shCtrl were seeded in 96-well plates at 2х10^4^ cells per well. Cell apoptosis was analyzed with a Caspase3/7 active cell apoptosis detection kit (Sangon Biotech, Shanghai, China). Caspase3/7 detection buffer was prepared by mixing 50 μl Caspase3/7 substrate and 10 ml assay buffer. Caspase3/7 detection buffer (100 μl) was added to each well of a 96-well plate and then incubated at room temperature in the dark for 1 h. The fluorescence intensity was detected at Ex/Em = 490/525 nm using flow cytometry (BD, Biosciences, USA).

### Statistical analysis

The statistical significance of differences this study was analyzed using GraphPad Prism 8. Comparisons between two groups were conducted through independent sample t tests, and comparisons between multiple groups were conducted through one-way ANOVA. All the experiments were repeated three independent times. P *<* 0.05 represented statistical significance.

### Electronic supplementary material

Below is the link to the electronic supplementary material.

**Supplementary Material 1: Figure S1** Detecting the knockdown efficiency of OIP5 after siRNA lentivirus infection by PCR. Among them, the sh3 target knockdown efficiency was the highest. Therefore, sh3 cells were used for subsequent experiments

**Supplementary Material 2: Figure S2** CCK8 assay showed that knocking down the OIP5 gene inhibited the proliferation of SKOV3 ovarian cancer cells

**Supplementary Material 3: Figure S3** Knockdown of OIP5 inhibited the migration and invasion of OVCAR-3 cells. A OIP5 silencing significantly inhibited the migration of OVCAR-3 cells. B Cell counting was performed on migrating cells. C The numbers of migrating cells in the shCtrl samples were set to 1, and the fold change was determined for the shOIP5 samples. D OIP5 silencing significantly inhibited the invasion of ovarian cancer cells. E Cell counting was performed on invading cells. F The numbers of invading cells in the shCtrl samples were set to 1, and the fold change was obtained for the shOIP5 samples. **P < 0.01. ****P < 0.0001

**Supplementary Material 4: Table S1** OIP5 lentivirus interference target design results

## Data Availability

The original contributions presented in this study are included in the article/Supplementary material, further inquiries can be directed to the corresponding authors.

## Abbreviations

ACC: Adrenocortical carcinoma
BLCA: Bladder Urothelial Carcinoma
BRCA: Breast invasive carcinoma
CESC: Cervical squamous cell carcinoma and endocervical adenocarcinoma
CHOL: Cholangiocarcinoma
COAD: Colon adenocarcinoma
ESCA: Esophageal carcinoma
GBMLGG: Glioma
GEO: Gene Expression Omnibus
GEPIA: Gene Expression Profiling Interactive Analysis
GO: Gene Ontology
GTEx: Genotype-Tissue Expression
HNSC: Head and Neck squamous cell carcinoma
KIRC: Kidney renal clear cell carcinoma
LIHC: Liver hepatocellular carcinoma
LUAD: Lung adenocarcinoma
LUSC: Lung squamous cell carcinoma
OIP5: Opa interacting protein 5
OV: Ovarian cancer
OV: Ovarian serous cystadenocarcinoma
PAAD: Pancreatic adenocarcinoma
PRAD: Prostate adenocarcinoma
ROC: Receiver operating characteristic
STAD: Stomach adenocarcinoma
STES: Stomach and Esophageal carcinoma
TCGA: The Cancer Genome Atlas
TGCT: Testicular Germ Cell Tumors
UCEC: Uterine Corpus Endometrial Carcinoma
