# Supplementary figures and images for: The role of OIP5 in the carcinogenesis and progression of ovarian cancer
Source: J Ovarian Res. 2023 Sep 2;16:185. doi: 10.1186/s13048-023-01265-4 (PMC10474646; doi:10.1186/s13048-023-01265-4)

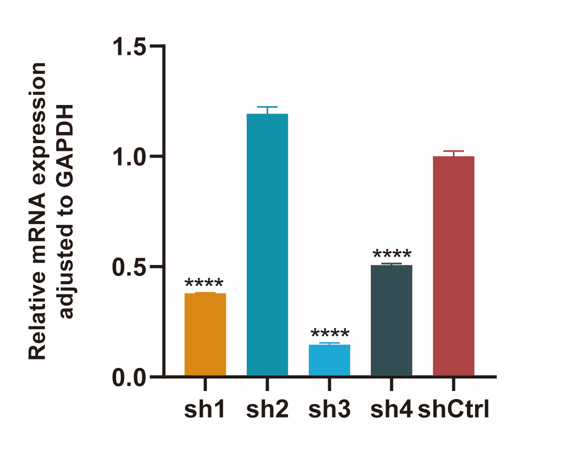

Supplement: Supplementary file 1 — Supplementary Material 1: Figure S1 Detecting the knockdown efficiency of OIP5 after siRNA lentivirus infection by PCR. Among them, the sh3 target knockdown efficiency was the highest. Therefore, sh3 cells were used for subsequent experiments [file 13048_2023_1265_MOESM1_ESM.png]

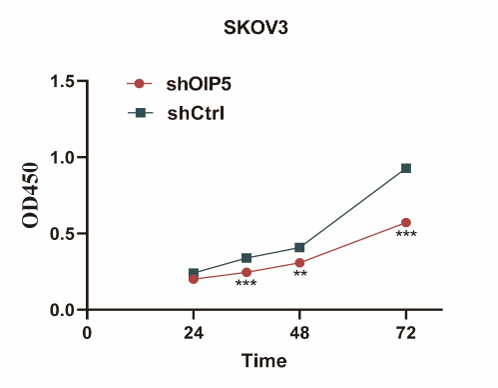

Supplement: Supplementary file 2 — Supplementary Material 2: Figure S2 CCK8 assay showed that knocking down the OIP5 gene inhibited the proliferation of SKOV3 ovarian cancer cells [file 13048_2023_1265_MOESM2_ESM.png]

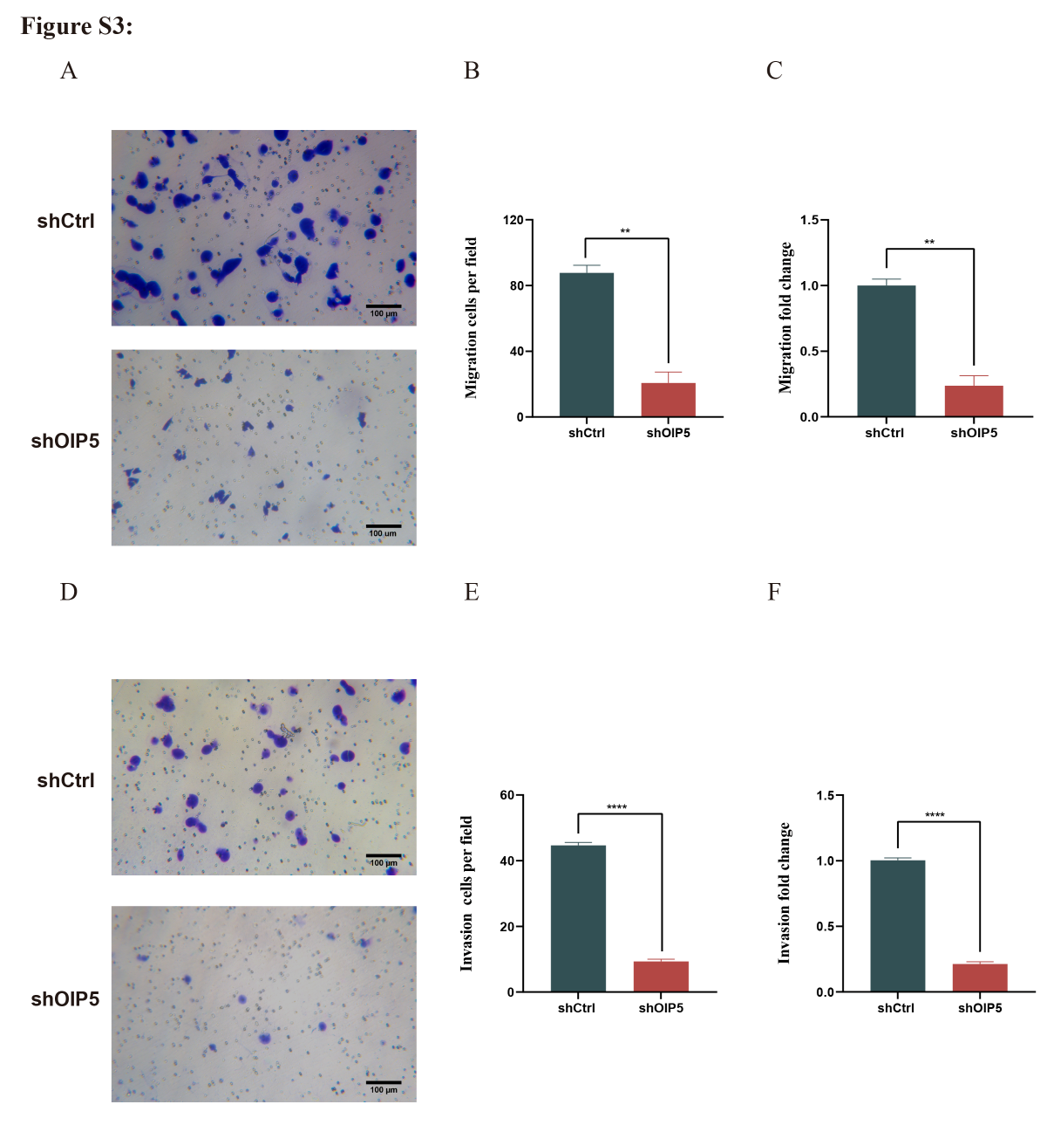

Supplement: Supplementary file 3 — Supplementary Material 3: Figure S3 Knockdown of OIP5 inhibited the migration and invasion of OVCAR-3 cells. A OIP5 silencing significantly inhibited the migration of OVCAR-3 cells. B Cell counting was performed on migrating cells. C The numbers of migrating cells in the shCtrl samples were set to 1, and the fold change was determined for the shOIP5 samples. D OIP5 silencing significantly inhibited the invasion of ovarian cancer cells. E Cell counting was performed on invading cells. F The numbers of invading cells in the shCtrl samples were set to 1, and the fold change was obtained for the shOIP5 samples. **P < 0.01. ****P < 0.0001 [file 13048_2023_1265_MOESM3_ESM.png]
