# Supplementary material for: The role of OIP5 in the carcinogenesis and progression of ovarian cancer
Source: J Ovarian Res. 2023 Sep 2;16:185. doi: 10.1186/s13048-023-01265-4 (PMC10474646; doi:10.1186/s13048-023-01265-4)
Supplement: Supplementary file 4 — Supplementary Material 4: Table S1 OIP5 lentivirus interference target design results [file 13048_2023_1265_MOESM4_ESM.docx]

| OIP5 target 1(sh1)  OIP5 target 2(sh2)  OIP5 target 3(sh3)  OIP5 target 4(sh4) | AGGGCGATTGACCAAGCTTCT  GCCCTTCCTAGTTGGCATTGA  GCATTGAAGGTTCACTCAAAG  GGTTCACTCAAAGGCAGCTAT |
| --- | --- |

**TableS1:** OIP5 Lentivirus interference target design results
